# Concentric and Eccentric Time-Under-Tension during Strengthening Exercises: Validity and Reliability of Stretch-Sensor Recordings from an Elastic Exercise-Band

**DOI:** 10.1371/journal.pone.0068172

**Published:** 2013-06-25

**Authors:** Michael Skovdal Rathleff, Kristian Thorborg, Thomas Bandholm

**Affiliations:** 1 Orthopaedic Surgery Research Unit, Aalborg University Hospital, Aalborg, Denmark; 2 Arthroscopic Centre Amager, Copenhagen University Hospital, Amager, Copenhagen, Denmark; 3 Department of Orthopedic Surgery, Copenhagen University Hospital, Hvidovre, Denmark; 4 Department of Physical Therapy, Physical Medicine and Rehabilitation Research – Copenhagen, Copenhagen University Hospital, Hvidovre, Denmark; 5 Clinical Research Centre, Copenhagen University Hospital, Hvidovre, Copenhagen, Denmark; The University of Queensland, Australia

## Abstract

**Background:**

Total, single repetition and contraction-phase specific (concentric and eccentric) time-under-tension (TUT) are important exercise-descriptors, as they are linked to the physiological and clinical response in exercise and rehabilitation.

**Objective:**

To investigate the validity and reliability of total, single repetition, and contraction-phase specific TUT during shoulder abduction exercises, based on data from a stretch-sensor attached to an elastic exercise band.

**Methods:**

A concurrent validity and interrater reliability study with two raters was conducted. Twelve participants performed five sets of 10 repetitions of shoulder abduction exercises with an elastic exercise band. Exercises were video-recorded to assess concurrent validity between TUT from stretch-sensor data and from video recordings (gold standard). Agreement between methods was calculated using Limits of Agreement (LoA), and the association was assessed by Pearson correlation coefficients. Interrater reliability was calculated using intraclass correlation coefficients (ICC 2.1).

**Results:**

Total, single repetition, and contraction-phase specific TUT – determined from video and stretch-sensor data – were highly correlated (r>0.99). Agreement between methods was high, as LoA ranged from 0.0 to 3.1 seconds for total TUT (2.6% of mean TUT), from -0.26 to 0.56 seconds for single repetition TUT (6.9%), and from -0.29 to 0.56 seconds for contraction-phase specific TUT (13.2-21.1%). Interrater reliability for total, single repetition and contraction-phase specific TUT was high (ICC>0.99). Interrater agreement was high, as LoA ranged from -2.11 to 2.56 seconds for total TUT (4.7%), from -0.46 to 0.50 seconds for single repetition TUT (9.7%) and from -0.41 to 0.44 seconds for contraction-phase specific TUT (5.2-14.5%).

**Conclusion:**

Data from a stretch-sensor attached to an elastic exercise band is a valid measure of total and single repetition time-under-tension, and the procedure is highly reliable. This method will enable clinicians and researchers to objectively quantify if home-based exercises are performed as prescribed, with respect to time-under-tension.

## Introduction

In the rehabilitation of patients, home-based exercises using elastic exercise bands are often prescribed after a thorough initial instruction by the prescribing physician or physiotherapist [1–3]. Elastic exercise bands have several qualities that make them preferable in rehabilitation and clinical settings. They provide adjustable resistance, and have been shown to be effective in clinical trials on shoulder, neck, knee and hip pain [4–7]. However, adherence to home-based exercise programmes often appears to be insufficient [5,8–11].

We recently showed that automatically stored data from a stretch-sensor attached to a standard elastic exercise band could be used to accurately identify training adherence and quality of shoulder-abduction strength training [12]. In the current study, we used the same technology to investigate if stored data from the stretch sensor could be used to quantify time-under-tension (TUT) of shoulder-abduction strength training.

Total TUT refers to the total time of all concentric, quasi-isometric and eccentric contraction-phases in a single training set [13]. Together with load and movement velocity it is an important strength training descriptor, as it reflects the time factor of the strength training stimulus [13–15]. Physiologically, greater TUT has been shown to increase myofibrillar protein synthesis more than lesser TUT after a single, work-matched, strength training session in healthy subjects [16]. Contraction-phase specific TUT refers to the time of the individual phases of TUT (concentric, quasi-isometric, and eccentric contraction-phases) in a single repetition [13,17]. Specific contraction-phases including their TUT are also important strength training descriptors [13,17]. Physiologically, evidence from animal studies suggests that eccentric contractions may govern specific mechanisms responsible for tendon healing, while isolated concentric contractions may not have similar effects [18–20]. However, clinical, evidence suggest that the combination of concentric and eccentric contractions may be more effective than eccentric contractions alone on the clinical outcome in achilles and patellar tendinopathy [21]. In healthy subjects, eccentric contractions elicit larger increases in strength and hypertrophy compared to concentric contractions [22]. Hence, the quantification of both total and contraction-phase specific TUT of performed strength training of the shoulder abductors is important in order to determine if executed training constitutes a sufficient physiological and clinical stimulus. In particular, this is of importance if exercises are performed at home after initial instruction by a physician or physiotherapist. Simple tools capable of measuring TUT during exercises with elastic exercise bands are currently unavailable.

The objective of the study was therefore to investigate the validity and reliability of total, single repetition and contraction-phase specific TUT during shoulder abduction exercises, based on data from a simple set-up that included a stretch-sensor attached to a standard elastic exercise band.

## Methods

### Ethics statement

**Figure 1:**
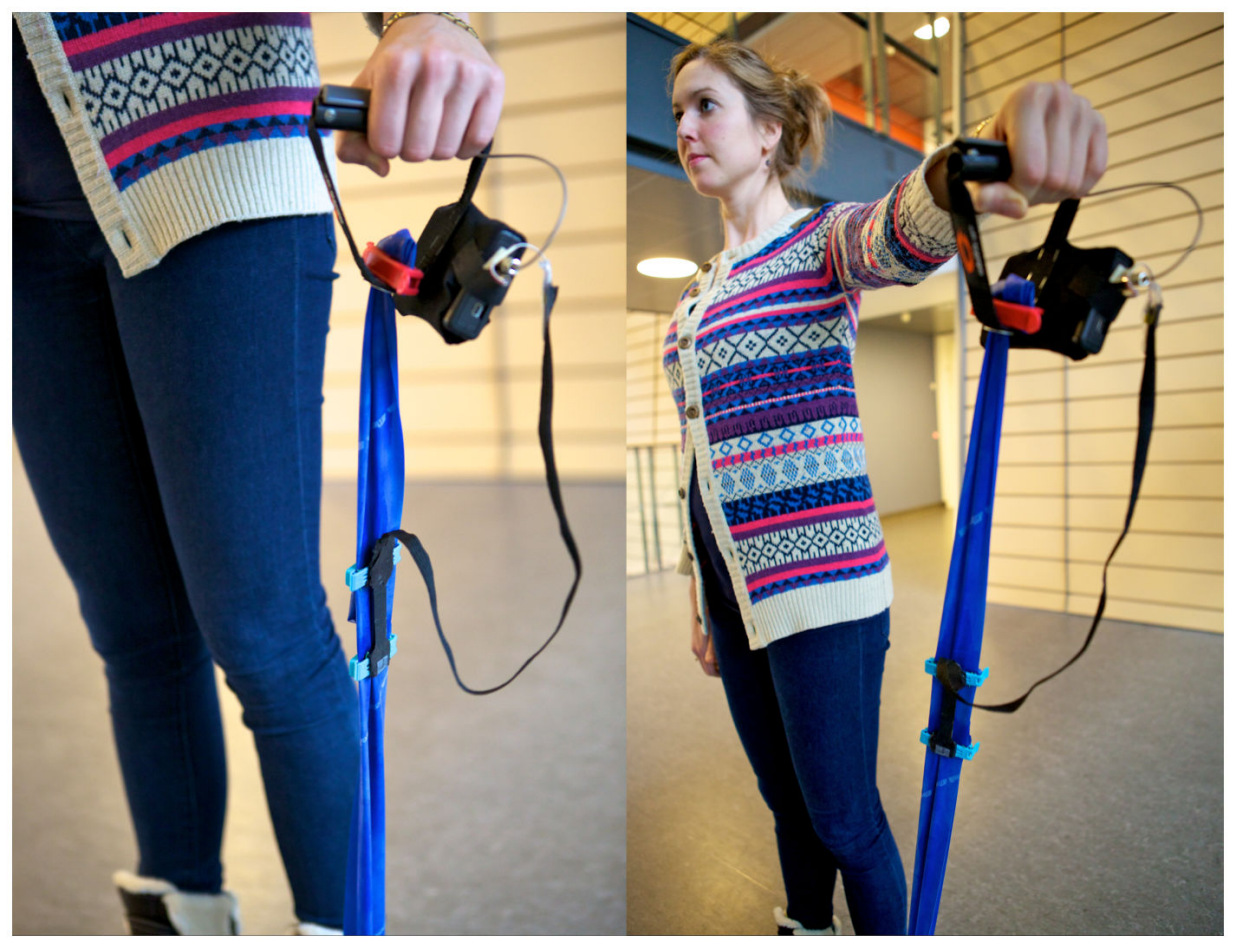
Elastic exercise band with the stretch sensor attached.

**Figure 2:**
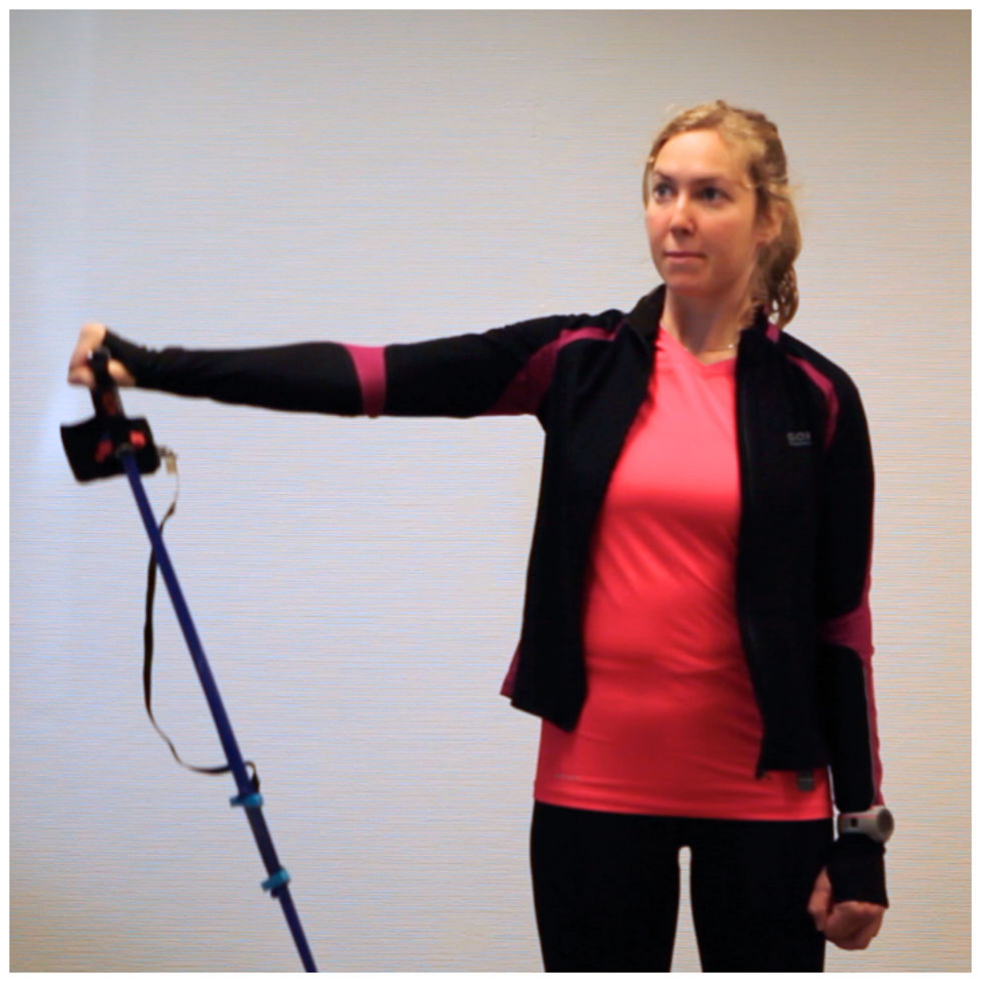
Image showing a screen-shot from the video recordings.

All participants were provided with verbal and written information about the procedures of the study, and written informed consent was obtained in accordance with the Declaration of Helsinki. The Ethics Committee in the North Denmark Region and the Danish Data Agency approved the study (2012-2410). The participant in Figures 1 and 2 has given written informed consent, as outlined in the PLOS consent form, for publication of their photograph.

### Design

The study was a concurrent validity and interrater reliability study. The study investigated if it were possible to determine total TUT, single TUT and contraction-phase specific TUT during shoulder abduction exercises, based on data recordings from a stretch-sensor attached to an elastic exercise band. Video recordings were used as a gold standard, and recorded concordantly, so each rater could determine, from both video recordings and stretch sensor data, the total TUT, single repetition TUT and contraction-phase specific TUT. The reporting of the study follows the Guidelines for Reporting Reliability and Agreement Studies (GRRAS) [23].

### Participants

A convenience sample of 12 healthy volunteers from the local hospital staff was recruited. They were between 21 and 29 years of age and free of upper extremity symptoms.

### Raters

Two raters with different clinical and technological experience using elastic band exercise were recruited. Hence, we considered the two raters representative of a larger population of raters who, in a future setting, would use the stretch-sensor equipment being investigated. Rater 1 was a male research physiotherapist with three years of research experience and 10 hours of prior experience of rating stretch-sensor data. Rater 2 was a female physiotherapist with three years of clinical experience and no previous experience in rating stretch-sensor data. Rater 1 already had experience in rating video recordings and stretch-sensor data and received no additional practice. Rater 2 was given 30 minutes of practice in rating stretch-sensor data from participants who were not part of the current study sample.

## Equipment

### Stretch-sensor and elastic exercise band

The elastic stretch-sensor was based on technology designed by Danfoss PolyPower (Nordborg, Denmark) and custom-made for the application. It acts as an elastic capacitive material that is stretchable in one direction. To measure the change in capacitance of the stretch-sensor, an electrical circuit was built with a timer. The circuit was built in such a way that a change in capacitance of the stretch-sensor corresponded to a change in the timer frequency, which allowed us to measure how much the sensor was being stretched (for further details, see Kappel et al [24]). The sensor was attached to the rubber band by two clips that made the sensor easily transferable to other elastic exercise bands, Figure 1. The sensor was attached via an USB connector to a small box that contains the electrical circuit, timer and data logger. The sampling frequency of the box was 200 Hz. The sensor is robust, and the material properties are robust to changes during usage [24]. A switch was mounted in the handle of the elastic exercise band, so that the data recording would start whenever the handle was pressed. The elastic band was a standard blue Thera-Band exercise band, which is commonly used in rehabilitation studies [1,4,5,25]. Before the exercise data were collected, we tested the handle switch by pressing it a total of 100 times. Every time the handle was pressed, the data collection started as intended, and the correct information regarding date and time of day was stored on the memory card [12].

### The exercise

Participants were instructed in performing shoulder abduction exercises from 0 to 90 degrees abduction. They were told to perform 10 repetitions at a self-determined speed, meaning there was no restriction to perform either of the contraction-phases at a predetermined speed or at a relative loading (repetition maximum). However, we secured that no slack was present in the exercise band at 0 degrees abduction meaning that the tension in the elastic band increased throughout the shoulder abduction with a peak at the end of the abduction due to the elastic tension. This approach was chosen to ensure a wide range of different speeds of exercises. All participants performed five sets of 10 repetitions with a 2-minute break between each set. The exercises were performed in front of a white wall, and recorded with a 50mm lens on a Canon 5D Mark II mounted on a tripod, Figure 2. Video was recorded at a resolution of 1920x1080 pixels at 24 frames per second. Hence, the sampling frequency was different between the stretch-sensor and video recordings (200 Hz. vs. 24 Hz.) and not synchronised. However this should not pose a treat to the findings of the study, as the design resembles the validity studies performed on handheld dynamometry versus isokinetic dynamometry (gold standard) where synchronization is impossible and sampling frequency is different between methods.

## Rating

### Stretch-sensor data

**Figure 3:**
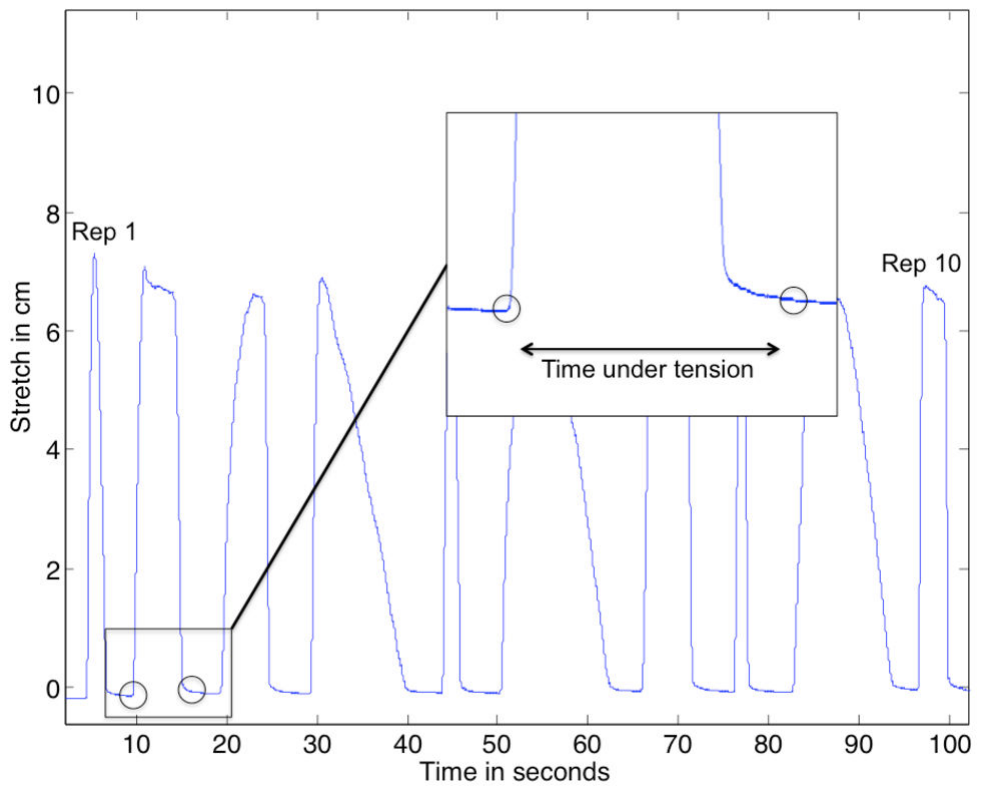
Ten repetitions of shoulder abduction. Typical example of data recordings from the stretch sensor. The starting and ending points of the repetition are indicated by the circles.

**Figure 4:**
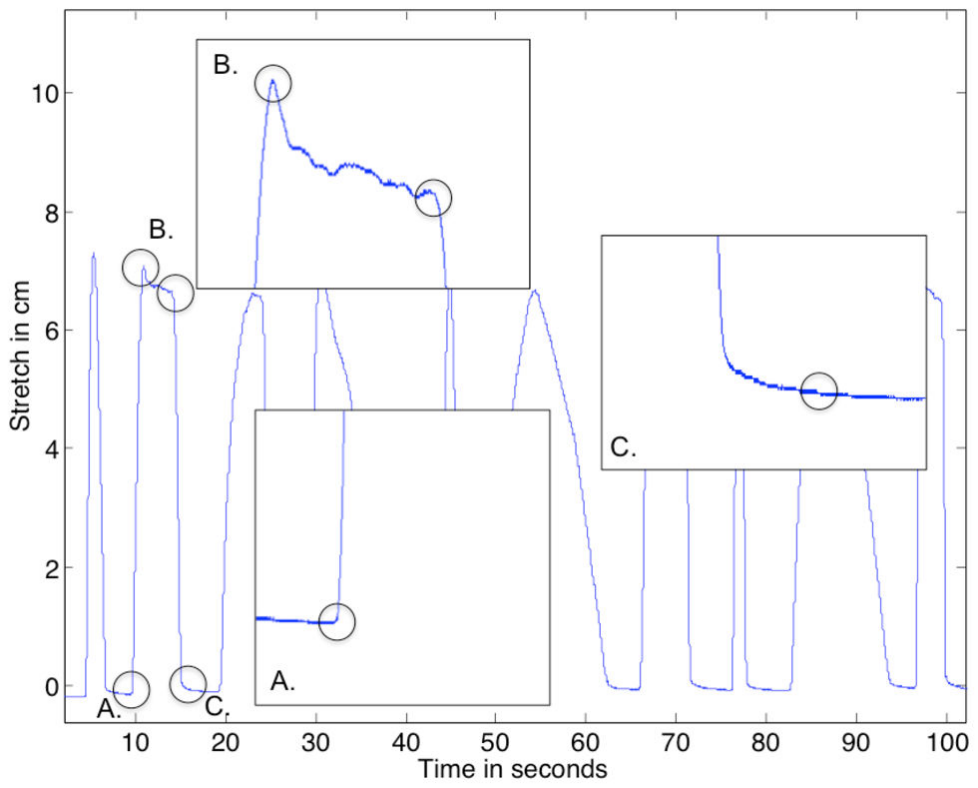
10 repetitions of shoulder abduction. For repetition 2, A illustrates the start of the concentric phase, B illustrates the end of concentric phase (start of the quasi-isometric phase, left circle) and the end of the quasi-isometric phase (start of the eccentric phase, right circle), and C illustrates the eccentric phase and the gradual transition from the eccentric phase to pause at 0 degrees abduction.

Rating of the stretch sensor data was done by analysing data from the stretch-sensor with Matlab 2011a (The MathWorks, Nattick, USA). A custom-written Matlab programme transformed the stretch-sensor data into an image showing the stretch of the sensor as a function of time (see example in Figure 3, and Matlab code in Supporting Information, Appendix S1). Afterwards, the mouse cursor was manually used to select the visually observed time-points that corresponded to the contraction-specific phases. The start of the concentric phase was defined as the last data-point before the signal started to increase, see Figure 4. The end of the concentric phase (the start of the quasi-isometric phase) was defined as the first data-point after the signal stopped increasing. The end of the quasi-isometric phase (start of the eccentric phase) was defined as the last data-point before the rapid decrease of the signal. The end of the eccentric phase was defined as the first data point where there was no further decrease in the signal. In some participants, a gradual transition from the end of the eccentric phase to the pause at 0 degrees abduction was observed. In these cases, the end of the eccentric phase was defined as the first data point where the signal returned to the mean baseline value (visually determined) in between each repetition.

### Video recordings

The free software programme V1 Home Basic (www.v1golfacademy.com) was used for playback of the video recordings. The software programme allows for frame-by-frame playback of the video recordings and thereby a precise determination of contraction-phase specific time points. The starting point of the concentric phase was defined as the first frame where the hand holding the elastic exercise band moved in the direction of abduction. The end of the concentric phase (the start of the quasi-isometric phase) was defined as the first frame where the hand no longer moved. The end of the quasi-isometric phase (the start of the eccentric phase) was defined as the first frame where the hand moved in the direction of adduction. The end of the eccentric phase was defined as the first frame where the hand no longer moved.

The following variables were determined from both video and stretch sensor data: (I) Total TUT, defined as the sum of the concentric, quasi-isometric and eccentric TUT during each set (10 repetitions) [13], (II) single repetition TUT, defined as the duration of each single repetition [13], and (III) contraction-phase specific TUT, defined as the duration of the concentric and eccentric phases of each repetition, respectively [13]. Rating of stretch-sensor data and video recordings were timed to allow for a comparison of the time-requirements for both methods. Quasi-isometric TUT was not included in the analysis as subjects performed the shoulder abduction at a self-selected speed and most subjects did not perform an quasi-isometric contraction.

## Data and statistical analyses

### Analysis 1: Validity

This analysis was performed to determine if total TUT, single repetition TUT and contraction-phase specific TUT could be determined validly from data recorded by the stretch-sensor. TUT determined from the video recordings was used as the gold standard. One rater (Rater 1) determined the total, single-repetition and contraction-phase specific TUT from all five exercise sets from three randomly chosen participants, from both video recordings and stretch-sensor data. This corresponded to 15 exercise sets consisting of 150 contraction-specific phases per method for both concentric and eccentric phases. The rater was blinded as to which subject he was rating and which of the five exercise sets he was rating. Firstly, all rating of video recordings was done, followed by rating of the stretch-sensor data. During rating of the video recordings and stretch-sensor data, the rater was randomly presented with one of the five exercise sets from the three different participants, until all 15 exercise sets had been rated.

**Figure 5:**
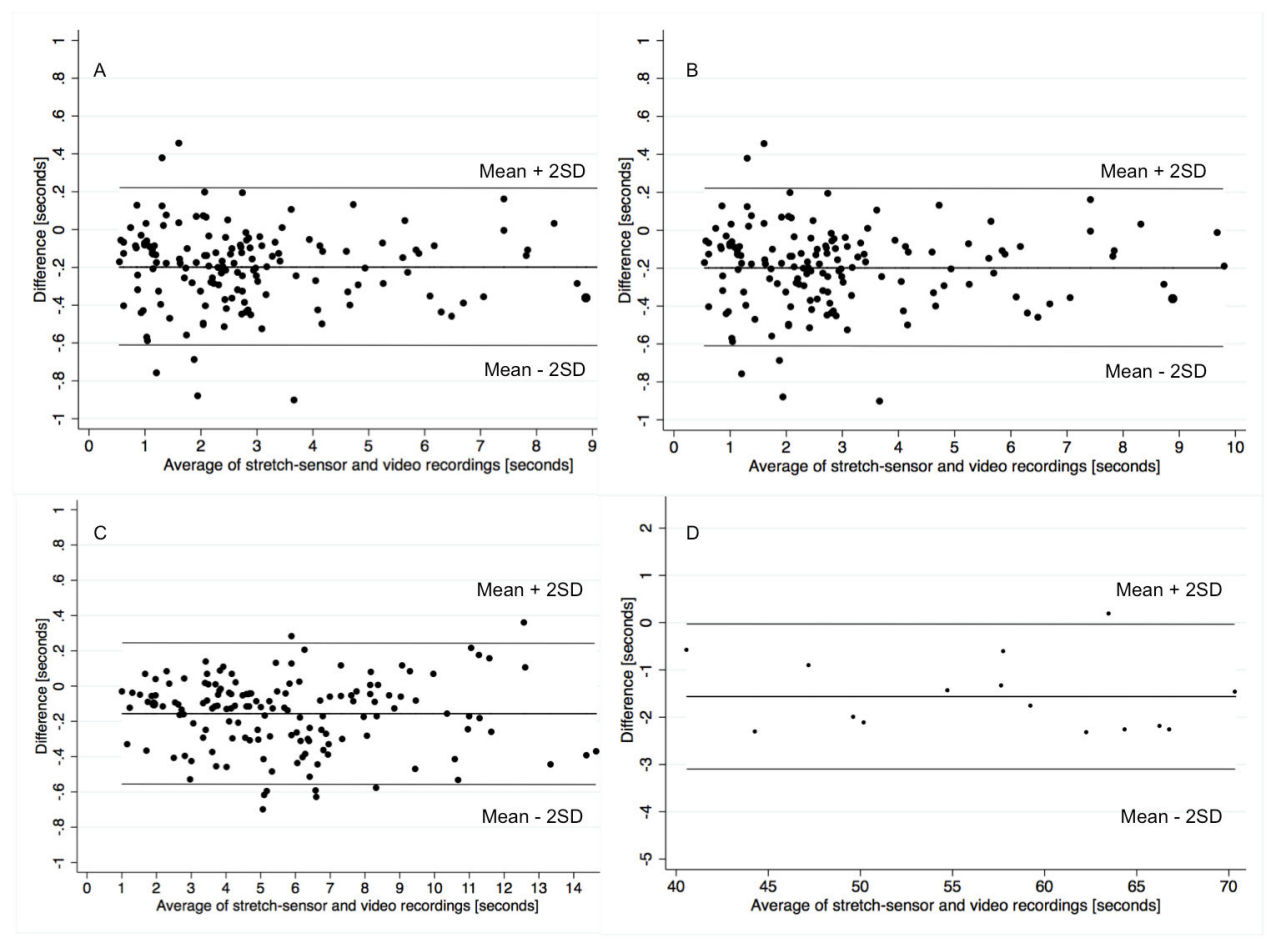
Bland-Altman plots showing the agreement between stretch-sensor recordings and video recordings. A: concentric contraction-phase. B: eccentric contraction-phase C: single repetition. D: total time-under-tension:.

For the statistical analyses of validity, paired t-tests were used to test for systematic bias between the two methods. Systematic bias is reported as the mean difference between methods and graphically presented as Bland-Altman plot in Figure 5. Pearson correlation coefficients were used to express the degree of linear association between the two methods. Finally, Limits of Agreements (LoA) were used to express the agreement between the two methods [26]. The LoA were calculated as the mean difference between methods ±1.96 times the standard deviation of the differences in total, single repetition and contraction-phase specific TUT between stretch-sensor data and video recordings. The LoA were presented as a range indicating the maximal potential difference between the two methods in 95% of the ratings and relative agreement was calculated as mean TUT divided by LoA [26]. Heteroscedasticity was visually assessed and there we no trends towards heteroscedasticity.

### Analysis 2: Interrater reliability of stretch-sensor data

This analysis was performed to determine the interrater reliability and agreement between independent raters, rating the stretch-sensor data. Raters 1 and 2 rated all 60 exercise sets from all 12 participants and determined TUT for each of the 600 single repetitions.

For the statistical analysis, a two-way random effects model (2.1), single measures, absolute agreement, and intraclass correlation coefficients (ICC) were used to express interrater reliability, while LoA were used to express agreement between raters [26]. LoAs were calculated and presented in the same way as in the validity analysis, but based on the difference in TUT between raters. The reliability analysis included total TUT for all 10 repetitions (n=60), total concentric and eccentric contraction-phase specific TUT for all 10 repetitions in each exercise set (n=60), single repetition TUT (n=600) and contraction-specific TUT for all repetitions (n=600).

### Sample size

We used a non-inferiority sample size calculation to determine the sample-size required for the validity analysis. This approach was chosen, as the primary goal of the validity analysis was to show that there were no clinically relevant differences between total TUT determined from video recordings and stretch-sensor data. The data used for the sample-size calculation were collected in a pilot study. Using a non-inferiority limit of 2 seconds of total TUT (3.3% of the total TUT in the pilot study) and a standard deviation of 1.5 seconds at 5% significance and 80% power, it was necessary to rate 14 exercise sets. We chose to increase the number of exercise sets to 15, as this enabled us to use all exercise sets from three randomly selected participants. The sample-size for the reliability analysis included 60 data points for total TUT, 60 data points for total concentric and eccentric contraction-phase and 600 data points for single repetition TUT and contraction specific TUT [27].

## Results

### Analysis 1: Validity

Total TUT, single repetition TUT and contraction-phase specific TUT-determined from video recordings and stretch sensor data – were highly correlated (r>0.99). Moreover, the agreement between the two methods was high, as the LoA ranged from 0.0 to 3.1 seconds for total TUT (2.6% of mean TUT), from -0.26 to 0.56 seconds for single repetition TUT (6.9% of mean TUT), and from -0.29 to 0.56 seconds for contraction-phase specific TUT (13.2-21.1% of mean TUT), Table 1 Figure 5. Generally, TUT determined from stretch sensor data was systematically of longer duration, compared with those determined from video recordings (p < 0.00001). These systematic differences ranged from 1.56 seconds for total TUT (2.8%), 0.16 seconds (2.8%) for single repetition TUT and from 0.09–0.20 seconds (4.7-6.9%) for contraction-phase specific TUT. The differences for total TUT were less than that defined as the non-inferiority limit of 2 seconds for the total TUT.

**Table 1:** Agreement between total, single repetition, and contraction-phase specific time-under-tension, determined from video recordings and stretch-sensor data.

Rater 3	Mean time based on stretch-sensor data. Seconds (±SD)	Mean time based on video recordings. Seconds (±SD)	Mean difference, seconds (95% CI)	Correlation	95% Limits of agreement (seconds)	Agreement (percentage of mean TUT from stretch-sensor)
Total TUT (n=15)	57.77 (9.02)	56.2 (8.91)	1.56 (1.13-2.00)*	1.00*	0.03-3.09	2.6%
Single repetition TUT (n=150)	5.78 (2.93)	5.62 (2.92)	0.16 (0.12-0.19)*	1.00*	-0.26-0.56	6.9%
Concentric TUT (n=150)	1.91 (1.30)	1.82 (1.28)	0.09 (0.06-0.11)*	0.99*	-0.22-0.40	16.2%
Eccentric TUT (n=150)	3.10 (2.07)	2.90 (2.07)	0.20 (0.16-0.23)*	0.99*	-0.21-0.61	13.2%

Abbreviations: TUT: Time-under-tension

* p<0.00001

### Analysis 2: Interrater reliability

The ICC for interrater reliability for total, single repetition and contraction-phase specific TUT was above 0.99, Table 2. The agreement between raters for total TUT ranged from -2.11 to 2.56 seconds (4.7% of mean TUT). The agreement for single repetitions was between -0.46 to 0.50 seconds (9.7% of mean TUT) and from -0.41 to 0.44 seconds for the contraction phases (5.2-14.5% of mean TUT). There were small systematic differences between raters (p<0.05) which ranged from 0.02 seconds (0.4%) for single repetition TUT to 0.04 seconds (2.2%) at total concentric TUT.

**Table 2:** Interrater reliability and 95% limits of agreement between Raters 1 and 2 for total, single and contraction-phase specific time-under-tension.

	Mean rater 1 seconds (±SD)	Mean rater 2 seconds (±SD)	Mean difference between raters, seconds (95% CI)	Intraclass correlation (95% CI)	95% Limits of agreement (seconds)	Agreement (percentage of mean TUT from stretch-sensor)
Total TUT (n=60)	49.28 (12.9)	49.50 (13.2)	-0.23 (-0.53-0.08)**	1.00 (0.99-1.00)	-2.11-2.56	4.7%
Single repetition TUT (n=600)	4.93 (2.11)	4.95 (2.14)	-0.02 (-0.04-0.00)*	0.99 (0.99-0.99)	-0.46-0.50	9.7%
Total concentric TUT (n=60)	17.66 (5.30)	18.06 (5.39)	-0.40 (-0.52--0.28)*	0.99 (0.96-1.00)	-1.32-0.51	5.2%
Total eccentric TUT (n=60)	29.02 (7.23)	28.87 (7.41)	0.15 (-0.15-0.45)**	0.99 (0.98-1.00)	-2.40-2.10	7.7%
Concentric TUT (n=600)	1.77 (1.02)	1.81 (1.02)	-0.04 (-0.05--0.03)*	0.99 (0.99-0.99)	-0.28-0.10	13.6%
Eccentric TUT (n=600)	2.90 (1.43)	2.89 (1.43)	0.02 (0.00-0.03)**	0.99 (0.99-0.99)	-0.41-0.44	14.5%

Abbreviations: TUT: Time-under-tension

* p<0.05

** p>0.05

### Time requirements for rating video recordings and stretch-sensor recordings

Both contraction-phase specific TUT and total TUT from video recordings took significantly longer time to calculate compared to calculating TUT when using stretch-sensor data (p<0.0001). Rating the video recordings of 10 repetitions of contraction-phase specific TUT took, on average, 17: 35 minutes. In comparison, it took, on average, 55 seconds to determine total TUT for 10 repetitions from stretch-sensor recordings and 3: 10 minutes to rate contraction-phase specific TUT for 10 repetitions.

## Discussion

Currently, there are no tools available to determine time-under-tension during home-based exercises, performed using elastic exercise bands. Such a tool is especially relevant, as patients most often perform home-based exercises alone, without being under the surveillance of the prescribing physician or therapist. Therefore, we investigated if data from a new stretch-sensor attached to a standard elastic exercise band could be used to validly and reliably calculate total TUT, single TUT and contraction-phase specific TUT. The current data show that total and single repetition TUT can be validly and reliably determined from stretch-sensor data. We previously validated the ability of this tool to identify specific exercise scenarios with different ranges of motion and contraction speeds when subjects performed dynamic shoulder abductions at a relative intensity of 12 repetition maximum (RM). Combining TUT with range of motion, and relative intensity determined by a test of repetitions maximum allows for a detailed description of the specific strength training stimulus.

### Practical relevance of the results

For illustration purposes, imagine two subjects, both enrolled in a research study investigating the effectiveness of a home-based strength-training intervention. Previous work indicates that total TUT in addition to concentric and eccentric contractions are important for the intervention to be able to induce a clinical effect [28,29]. The eccentric contractions, in particular, seem to be important [18,19]. The two subjects are both instructed to follow the exercise prescription of three training sessions per week. Each prescribed training session should consist of shoulder abduction from 0 degrees of abduction to 90 degrees of abduction in three sets of 10 repetitions at twelve RM. Each prescribed repetition should consist of a three seconds concentric phase, two seconds quasi-isometric phase at 90 degrees of shoulder abduction, and finally three seconds of eccentric phase.

Both patients adhere to the prescribed programme and relative load, but one patient does perform the exercises too fast; two seconds of concentric phase, no quasi-isometric phase at 90 degrees, and then a one second eccentric phase. After 12 weeks of the programme, both patients will have performed an identical number of exercise sets and repetitions and the patient who adhered to the prescribed TUT will have performed exercises corresponding to a TUT of 8640 seconds (144 minutes). However, the patient who did not adhere to the prescribed TUT, will have performed exercises corresponding to only 37.5% of the total training stimulus and 33.3% of the eccentric stimulus of the patient who adhered. It seems plausible that such variations in training volume (TUT) and training specificity (contraction-phase specific TUT) may lead to differences in the physiological and clinical response even though the same number of repetitions were performed by the two patients [16,28–30]. If exercise adherence were recorded using an exercise diary in the theoretical example above, both patients would have appeared equally adherent to the prescribed exercise programme.

If instead the patients had trained using the elastic exercise band and stretch-sensor investigated in the current study, the treating physician or physiotherapist could have measured TUT for sets, single repetitions and specific contraction-phases. Afterwards, it would have been possible to calculate how close the measured TUT corresponded to e.g. the prescribed eccentric TUT and thereby quantify adherence as a percentage of the prescribed total eccentric TUT during the programme.

Another way of measuring TUT during home-based exercises is to use webcams connected to a computer. This approach has been used in the field of tele-rehabilitation of patients with stroke [31,32]. However, this solution requires that patients remember to turn on the webcams before starting to exercise. Moreover, a computer and knowledge on how to operate it is mandatory. Even if a computer solution could be established, our data showed that determining TUT from video recordings is much more time-consuming than from stretch-sensor data. Accelerometer based solutions could also be used in some situations where the external load (for example a dumbbell) is known [33]. This allows for a detailed description of measuring force and power, which are also important strength training descriptors [13]. The stretch-sensor attached to an exercise-band seems to be extremely user-friendly, as it does not require specific equipment and skills, data collection is fully automatic and is exercise-integrated. In addition, it allows for identification of specific shoulder abduction exercise scenarios with different ranges of motion and contraction speeds when subjects performed dynamic shoulder abductions at a relative intensity of 12 repetition maximum (RM). Finally, and of great importance, the investigated method allow patients to perform prescribed exercises away from home, such as at work or when traveling.

### Improving reliability and agreement

All ratings were done manually, using the definition of concentric, quasi-isometric and eccentric phases, stated in “Methods”, and visualised in Figures 3 and 4. The transition from the end of the eccentric phase to the pause at 0 degrees abduction is often a gradual transition. In some cases, this makes it difficult to pinpoint the exact end of the eccentric phase. This may also be the reason why we observed the lowest reliability and agreement for single repetition eccentric TUT. However, some of this error is apparently reduced when summarising the single contraction TUT to TUT for a set of 10 repetitions, as the reliability for eccentric TUT in a set of 10 repetitions increases considerably. The clinical implication of this finding is that when using the stretch-sensor to investigate the adherence to prescribed eccentric TUT in physical medicine and rehabilitation, one should use the total eccentric TUT as opposed to evaluate each individual eccentric TUT for single repetitions.

A possible solution to increase reliability of the TUT of the eccentric phase could be to apply an automatic algorithm to detect the time spent in each of the three contraction-phases. One could also argue that the achieved degree of reliability and agreement using the manual rating procedure leaves little room for improvement, other than using the same rater for multiple ratings from the same patient. The reason for this is that intra-tester ratings are generally reported to be more reliable than inter-tester rating [34]. However, a likely benefit from using an automatic algorithm would be decreased time-requirements for rating data. The current manual detection took on average 3: 10min, while an automatic algorithm would likely decrease rating to a few seconds.

### Limitations

As a clinical gold standard for measuring TUT, we used video recordings with a sampling rate of 24 Hz. This is lower than the 200 Hz, used by the stretch sensor. It may have introduced some random error in the determination of the start and stopping points of the contraction phases, as the contraction may initiate between two image frames. The position transducers, used by Tran et al. [17] to measure TUT, may have introduced less random error. However, given the high degree of agreement between procedures found in the current study, any additional effect of using position transducers, as opposed to standard video recordings, would most likely not be of clinical relevance

### Future research

The example of the two patients above appearing to demonstrate equal adherence to the exercise programme, even though they display a large difference in total and eccentric TUT during an intervention period, suggests that future research should aim to describe adherence to exercise in more detail. Based on the research conducted in healthy subjects, showing that greater TUT induces a larger physiological stimulus, may hold true for patients as well. Using a stretch-sensor attached to an elastic exercise band would allow for similar studies to be conducted in various patient groups.

## Conclusion

Data from a stretch-sensor attached to an elastic exercise band can be used to calculate total and single repetition times under tension. The procedure is valid and highly reliable between raters. The current method will enable clinicians and researchers to objectively quantify if home-based exercises are performed correctly with respect to prescribed contraction phases and time-under-tension.

## Supporting Information

Appendix S1 Matlab code for generating images as seen in Figures 3 and 4. (DOCX)
