# Supplementary material for: Concentric and Eccentric Time-Under-Tension during Strengthening Exercises: Validity and Reliability of Stretch-Sensor Recordings from an Elastic Exercise-Band
Source: PLoS One. 2013 Jun 25;8(6):e68172. doi: 10.1371/journal.pone.0068172 (PMC3692465; doi:10.1371/journal.pone.0068172)
Supplement: Appendix S1 — (DOCX) [file pone.0068172.s001.docx]

Supporting Information

Appendix S1: Matlab code for generating images as seen in Figures 1 and 2.

filer = dir('./*.log')

N = 40*200; % number of samples to skip in end (30 sec x 200 samples/sec)

Ntotal = 20000; % full number of samples (extend if less)

T = 5000; % threshold - remove over this

dt = 1/200; % sample-time (200 Hz)

alp = 0.01; % strain-factor (calibration constant)

for i=1:length(filer),

filnavn = filer(i).name;

fid = fopen(filnavn);

fgetl(fid);

fgetl(fid);

fgetl(fid);

myArr = [];

while 1

tline = fgetl(fid);

if ~ischar(tline), break, end

myArr = [myArr ; str2num(tline)];

end

fclose(fid);

% find unstretched value

unStretch = mean(myArr(end-round(N/10):end));

% remove in end

myArr(end-N:end) = [];

% remove outliers (assume only a few of these..)

myArr(myArr>T) = [];

% plot + jpg-file

plot((1:length(myArr))*dt, -(myArr - unStretch)*alp)

title(filnavn(1:end-4))

xlabel('Time in seconds')

ylabel('Stretch in cm')

axis([0 100 -1 11]) % fixed x-axis length

% write jpeg-file

f = getframe(gcf);

imwrite(f.cdata, [filnavn(1:end-4) '.jpg'], 'jpeg')

end
